# Distinct Functional Contributions by the Conserved Domains of the Malaria Parasite Alveolin IMC1h

**DOI:** 10.3389/fcimb.2019.00266

**Published:** 2019-07-24

**Authors:** Michael P. Coghlan, Annie Z. Tremp, Sadia Saeed, Cara K. Vaughan, Johannes T. Dessens

**Affiliations:** ^1^Department of Infection Biology, London School of Hygiene and Tropical Medicine, London, United Kingdom; ^2^Institute of Structural and Molecular Biology, School of Biological Sciences, Birkbeck, London, United Kingdom

**Keywords:** *Plasmodium*, cytoskeleton, ookinete, sporozoite, motility, transmission

## Abstract

Invasive, motile life cycle stages (zoites) of apicomplexan parasites possess a cortical membrane skeleton composed of intermediate filaments with roles in zoite morphogenesis, tensile strength and motility. Its building blocks include a family of proteins called alveolins that are characterized by conserved “alveolin” domains composed of tandem repeat sequences. A subset of alveolins possess additional conserved domains that are structurally unrelated and the roles of which remain unclear. In this structure-function analysis we investigated the functional contributions of the “alveolin” vs. “non-alveolin” domains of IMC1h, a protein expressed in the ookinete and sporozoite life cycle stages of malaria parasites and essential for parasite transmission. Using allelic replacement in *Plasmodium berghei*, we show that the alveolin domain is responsible for targeting IMC1h to the membrane skeleton and, consequently, its deletion from the protein results in loss of function manifested by abnormally-shaped ookinetes and sporozoites with reduced tensile strength, motility and infectivity. Conversely, IMC1h lacking its non-alveolin conserved domain is correctly targeted and can facilitate tensile strength but not motility. Our findings support the concept that the alveolin module contains the properties for filament formation, and show for the first time that tensile strength makes an important contribution to zoite infectivity. The data furthermore provide new insight into the underlying molecular mechanisms of motility, indicating that tensile strength is mechanistically uncoupled from locomotion, and pointing to a role of the non-alveolin domain in the motility-enhancing properties of IMC1h possibly by engaging with the locomotion apparatus.

## Introduction

*Plasmodium* species, the causative agents of malaria, have a complex life cycle in vertebrate host and mosquito vector. Parasite-infected erythrocytes multiply via an asexual replication cycle called schizogony to release merozoites that infect new red blood cells. A small percentage of these develop into sexual stage precursor cells (gametocytes) which, after uptake with the blood meal of a feeding mosquito, begin a rapid process of gamete formation and fertilization inside the mosquito midgut. The resultant zygotes undergo meiosis and transform into elongated forms termed ookinetes, which traverse the midgut epithelium and then round up to form the oocysts. In the following weeks, young oocysts grow and divide by a process known as sporogony to generate hundreds of daughter cells named sporozoites. After egress from the oocyst, sporozoites invade and inhabit the salivary glands, and are transmitted to new hosts by mosquito bite to first infect liver cells from which new malaria blood stage infections are initiated to complete the life cycle.

The merozoite, ookinete, and sporozoite constitute the three motile and invasive stages in the *Plasmodium* life cycle. These so-called “zoite” stages possess a characteristic cortical structure termed the pellicle. The pellicle is defined by a double membrane structure termed the inner membrane complex (IMC) situated directly underneath the plasma membrane, which is equivalent to a sutured system of flattened sacs or alveoli (Bannister et al., 2000; Morrissette and Sibley, 2002; Santos et al., 2009). On the cytoplasmic face of the IMC, and tightly associated with it, sits a network of intermediate filaments termed the subpellicular network (SPN), a viscoelastic membrane skeleton that supports the IMC and provides tensile strength to the cell (Mann and Beckers, 2001). A family of IMC1 proteins, now called alveolins, have been identified as major components of the SPN (Mann and Beckers, 2001; Khater et al., 2004). Members of the alveolin family are found in apicomplexans and chromerids, as well as in ciliates and dinoflagellate algae, which together with the apicomplexans form the Alveolata superphylum (Gould et al., 2008). The alveolins are part of a larger class of proteins called epiplastins that aside alveolates have also been identified in euglenids, glaucophytes and cryptophytes (Goodenough et al., 2018). In the genus *Plasmodium*, 13 conserved and syntenic alveolin family members have thus far been identified that are differentially expressed among the three different zoites stages (Al-Khattaf et al., 2015; Kaneko et al., 2015). In addition, two PhIL1 interacting proteins: PIP2 and PIP3, show structural homology with alveolins (Kono et al., 2013; Parkyn Schneider et al., 2017).

It has been shown in the rodent malaria species *P. berghei* that disruption of the alveolins IMC1a, IMC1b, or IMC1h gives rise to morphological aberrations that are accompanied by reduced tensile strength of the zoite stages in which they are expressed. The same null mutant parasites also display motility defects, indicating that these alveolins also participate in parasite locomotion through an as yet unknown mechanism (Khater et al., 2004; Tremp et al., 2008; Tremp and Dessens, 2011; Volkmann et al., 2012). The SPN effectively separates the main cytosol from a smaller cortical cytoplasm that contains the molecular machinery that drives apicomplexan zoite motility, invasion and egress. Motility of apicomplexan zoites relies on an actinomyosin motor system that is situated in the space between the plasma membrane and the IMC. The conventional model is that the molecular motor and its auxiliary proteins is linked to cell surface adhesins via actin filaments and bridging proteins, and is internally anchored into the IMC. Motor force drives the actin filaments and adhesins rearward, thereby creating a traction force that propels the cell in the opposite direction against a substrate (Frenal et al., 2010). The IMC is underlain by the rigid yet flexible SPN, and this is most likely how alveolins assert their role in motility, either indirectly by providing mechanical support to the IMC, or through interactions with components of the motility apparatus.

The structural homologies between alveolin proteins are largely confined to conserved domains containing tandem repeat sequences (Al-Khattaf et al., 2015), herein referred to as “alveolin” domains. A subset of alveolins possess additional conserved modules that are structurally unrelated to the archetypal “alveolin” module. The roles of these “non-alveolin” domains in protein function are poorly understood. In this study, we investigated the functional contributions of the “alveolin” vs. “non-alveolin” modules of IMC1h, using the rodent malaria parasite species *P. berghei* and a strategy of allelic replacement and GFP tagging. IMC1h is expressed in both the ookinete and sporozoite life cycle stages of the parasite, where it carries out equivalent roles (Tremp and Dessens, 2011; Volkmann et al., 2012), thus allowing our investigations to be conducted across two distinct zoite stages. The results obtained indicate that the two IMC1h modules play distinct parts in facilitating morphogenesis, tensile strength and motility. The implications of these results are discussed in the context of parasite infectivity.

## Materials and Methods

### Animal Use

Experiments were conducted in 6–8 weeks old female CD1 mice, specific pathogen free and maintained in filter cages. Animal welfare was assessed daily and animals were humanely killed upon reaching experimental or humane endpoints. Mice were infected with parasites by intraperitoneal injection, or by infected mosquito bite on anesthetized animals. Parasitemia was monitored regularly by collecting of a small drop of blood from a superficial tail vein. Drugs were administered by intraperitoneal injection or where possible were supplied in drinking water. Parasitized blood was harvested by cardiac bleed under general anesthesia without recovery.

### Gene Targeting Vectors

**Figure 1:**
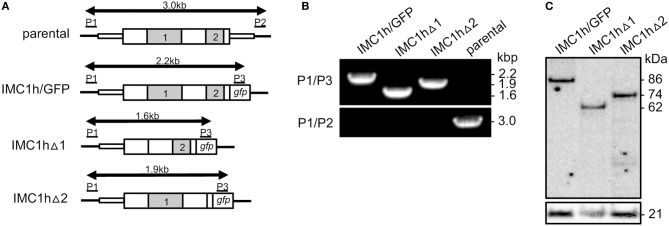
Generation and genotyping of IMC1h mutants. **(A)** Schematic diagram of the parental and modified *imc1h* alleles. The *imc1h* gene is indicated with coding sequence (wide bars) and 5′ and 3′ untranslated regions (narrow bars). The relative positions of domains 1 and 2 are indicated (gray). Also shown are the *gfp* module, positions of primers P1–P3 used for diagnostic PCR amplification, and the predicted lengths of the amplicons (arrows). **(B)** PCR with primers P1 and P3 diagnostic for integration of the modified, GFP-tagged *imc1h* alleles into the *imc1h* locus, or with primers P1 and P2 diagnostic for absence/presence of the unmodified parental *imc1h* allele. Amplicon sizes (kb) are indicated on the right-hand side and correspond to those depicted in **(A)**. **(C)** Western blot analysis of purified ookinetes (~ 50,000 per lane) of IMC1h mutant parasite lines using anti-GFP antibodies, showing successful expression of IMC1h::GFP fusion proteins. Identified band sizes reflect the introduced domain deletions in IMC1h, resulting in proteins of 86 kDa (IMC1h/GFP), 62 kDa (IMC1hΔ1), and 74 kDa (IMC1hΔ2). The ookinete surface protein Pbs21 is shown as a loading control.

To delete domain 1 of IMC1h (amino acids 103 to 306) primers IMC1hdeltadomain1-F (GAGGTTCACAATATTTGAATAACAATCAAGCACA) and IMC1hdeltadomain1-R (AAATATTGTGAACCTCCATACAAAGTGTGTT) were used to PCR amplify plasmid pLP-IMC1h/GFP (Tremp and Dessens, 2011). Template plasmid was removed after the PCR by *Dpn*I digestion, and the PCR product was circularized by in-fusion, to give plasmid pLP-IMC1hΔ1. This mutation deletes 205 amino acids from the IMC1h::GFP fusion protein (Figure 1). The same approach was used to delete domain 2 (amino acids 397 to 504), using primers IMC1hdeltadomain2-F (ATAATTCGGTTAAAGCTATCCAGAAAAACAT) and IMC1hdeltadomain2-R (GCTTTAACCGAATTATTTTGTCTATAATCCATATTTGA) to give plasmid pLP-IMC1hΔ2. This mutation deletes 109 amino acids from the IMC1h::GFP fusion protein (Figure 1).

### Generation and Genotyping of Genetically Modified Parasites

Parasite transfection, pyrimethamine selection and dilution cloning were performed as previously described (Waters et al., 1997). Prior to performing transfections, plasmid DNA was double-digested with KpnI and SacII to remove the plasmid backbone. Genomic DNA extraction was performed as previously described (Dessens et al., 1999). After transfection, drug resistant parasites were subjected to limiting dilution cloning. Integration of the GFP-tagged IMC1h-encoding sequence into the *imc1h* locus was confirmed by diagnostic PCR across the 5' integration site with primers P1 (CCATTTTTATGTTGAGCTTGAG) and P3 (GTGCCCATTAACCATCACC) (Figure 1). The absence of the unmodified *imc1h* allele in the clonal parasite lines was confirmed by diagnostic PCR with primers P1 and P2 (TTCTATAATCTTTAATTGTTCAGAAATGTG) (Figure 1).

### Sporozoite Size Measurements

Images of individual midgut sporozoites were captured by microscopy on Zeiss LSM510 inverted laser scanning confocal microscope. Using Zeiss LSM image browser software the circumference was measured from which the occupied surface area (“footprint”) was calculated as a measure of cell size. Statistical analysis was carried out using Student's *t*-test.

### Zoite Motility

Ookinete motility was assessed essentially as previously described (Moon et al., 2009). Aliquots of neat ookinete cultures were added to equal volumes of Matrigel (BD Biosciences) on ice, mixed thoroughly, spotted onto a microscope slide, and covered with a cover slip. After sealing with nail varnish, the Matrigel was allowed to set at room temperature for 30 min before analysis. Sporozoites were gently released from ~ 20 salivary glands in 200 μl RPMI medium in a Dounce homogenizer on ice. Following addition of an equal volume of RPMI supplemented with 20% fetal bovine serum, the sporozoites were transferred to an Eppendorf tube and collected by centrifugation in a swing-out rotor for 10 min at 1,000 × g at 4°C, followed by removal of excess supernatant. Aliquots of resuspended sporozoites were mixed with an equal volume of Matrigel on ice before transfer to microscope slides. After sealing the cover slip with nail varnish, the Matrigel was allowed to set at room temperature for 30 min before analysis. Cells were examined and time-lapse images taken on a Zeiss Axioplan II microscope. Movies were analyzed with ImageJ using the Manual Tracking plugin. Statistical analysis was carried out using ANOVA and Tukey's multiple comparison.

### Osmotic Shock and Viability Assays

Ookinetes in neat culture were subjected to hypo-osmotic shock of 0.5 × normal osmotic strength by adding an equal volume of water. Sporozoites were released from oocyst-infected midguts at 15 days post-infection and were subjected to 0.33 × normal osmotic strength by adding two equal volumes of water. After 5 min, normal osmotic conditions were restored by adding an appropriate amount of 10 × PBS. Cell viability was scored by fluorescence microscopy in the presence of 0.5% propidium iodide and 1% Hoechst 33258. Ookinetes whose nuclei stained positive for both propidium iodide and Hoechst were scored as non-viable, whereas ookinetes whose nuclei stained positive only for Hoechst were scored as viable. Values were normalized to 100% viability in untreated cells.

### Mosquito Infection

At 6 days before infecting mosquitoes, mice were injected intraperitoneally with phenylhydrazine (6 mg/ml in PBS, 10 μl/g body weight) to induce reticulocytosis. At 3 days before mosquito feeding, mice were infected intraperitoneally with 10^7^ parasitized red blood cells. The day of the feed, parasitemia and gametocytemia were checked by using a Giemsa-stained blood film. Mice were anesthetized and placed on a cage containing up to 50 starved female mosquitoes. Insects were allowed to blood feed in a draft-free, darkened environment at room temperature for 15 min. The day after feeding, unfed or partially fed mosquitoes (i.e., those unlikely to be infected) were removed if desired. For sporozoite transmission, the prevalence of infection of mosquito batches was determined and combined with the number of blood meal-positive insects after feeding to estimate the number of sporozoite-infected mosquitoes that fed.

## Results

### Generation and Genotyping of IMC1h Mutants

Multiple alignment of amino acid sequences of IMC1h orthologs from different *Plasmodium* species clearly reveals the presence of its single conserved “alveolin” module (domain 1), as well as a conserved carboxy-terminal module that is structurally unrelated (domain 2) (Tremp and Dessens, 2011). We previously generated and characterized parasites lines stably expressing IMC1h::GFP from the native *imc1h* promoter (named IMC1h/GFP) (Tremp and Dessens, 2011). To study the role of domains 1 and 2 we used the same allelic replacement strategy, generating transgenic parasites lines that express IMC1h::GFP without domain 1 (named IMC1hΔ1) or IMC1h::GFP lacking domain 2 (named IMC1hΔ2) (Figure 1A). Diagnostic PCR with primers P1 and P3 (Figure 1A) of clonal parasite lines amplified expected products of ~ 2.2, 1.6, and 1.9 kb from parasite lines IMC1h/GFP, IMC1hΔ1, and IMC1hΔ2, respectively, confirming integration of the modified *imc1h* alleles into the *imc1h* locus (Figure 1B). Moreover, amplification with primers P1 and P2 (Figure 1A) amplified an ~ 3 kb product only from the parental parasite, confirming absence of the unmodified *imc1h* allele in the transgenic lines (Figure 1B). Western blot analysis of purified ookinete samples using anti-GFP antibodies showed comparable expression levels of IMC1h::GFP fusion proteins in all three parasite lines, with protein sizes of the IMC1h::GFP fusion proteins corresponding to the introduced amino acid deletions in IMC1h (Figure 1C). This indicated that the truncated IMC1h proteins are stably expressed in ookinetes and confirms the successful introduction of the domain 1 and domain 2 deletions.

### Ookinete-Specific Subcellular Localization of Mutant IMC1h::GFP and Cell Shape

**Figure 2:**
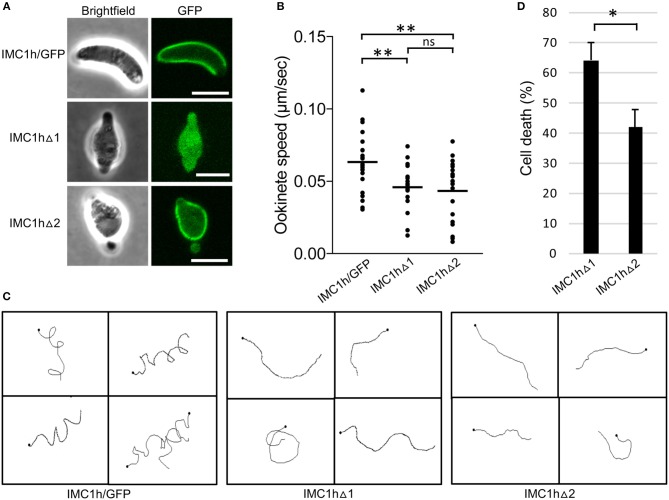
Ookinete-specific subcellular localization of IMC1h::GFP in IMC1h mutants, and their phenotypes. **(A)** Confocal GFP fluorescence and brightfield images of ookinetes of IMC1h mutant parasite lines. Note that IMC1hΔ2 ookinetes have abnormal shape, but display normal cortical localization of the GFP fusion protein. Scale bar = 5 μm. **(B)** Ookinete speed expressed in μm/sec. Images were captured every 10 s over a 10 min period. Plot is based on pooled data from two independent experiments and >60 ookinetes analyzed. Horizontal lines mark mean values. ^**^statistically significant differences (*P* < 0.05). ns, not significant. **(C)** Representative images of reconstructed 3D movement patterns from time-lapse movies of IMC1h mutants in Matrigel. The trajectories reveal the much straighter movement of IMC1hΔ1 and IMC1hΔ2 ookinetes compared to the helical movement of IMC1h/GFP ookinetes. **(D)** Percentage cell death after hypo-osmotic shock (0.5 × normal strength for 5 min). Cell death inversely correlates to tensile strength. ^*^statistically significant differences (*P* < 0.05). Error bars indicate S.E.M. values from four independent experiments.

To study the subcellular localization of the truncated IMC1h::GFP fusion proteins in parasite lines IMC1hΔ1 and IMC1hΔ2, live ookinetes were examined for GFP fluorescence. As described previously (Tremp and Dessens, 2011), ookinetes expressing full-length IMC1h::GFP had normal shape and displayed a predominantly cortical localization of GFP fluorescence (Figure 2A), consistent with the recruitment of IMC1h to the SPN. Both IMC1hΔ1 and IMC1hΔ2 ookinetes were misshapen, lacking the typical crescent shape and possessing a bulging area in the center (Figure 2A) similar to the shape of IMC1h-KO ookinetes (Tremp and Dessens, 2011). This indicates that domain 1 and domain 2 are both required for IMC1h to facilitate normal ookinete morphogenesis. Subcellular localization of GFP fluorescence was markedly different between IMC1hΔ1 and IMC1hΔ2 ookinetes: while the GFP signal in IMC1hΔ2 ookinetes was predominantly found at the cell cortex like in IMC1h/GFP ookinetes, IMC1hΔ1 ookinetes displayed only cytoplasmic fluorescence (Figure 2A). These observations show that domain 1 is required for recruitment of IMC1h to the SPN.

### Motility, Tensile Strength, and Infectivity of IMC1h Mutant Ookinetes

As the truncated IMC1h::GFP fusion protein expressed in IMC1hΔ1 ookinetes fails to reach its site of action: the SPN (Figure 2A), this parasite is effectively an IMC1h null mutant. Indeed, assessment of ookinete motility in Matrigel revealed that IMC1hΔ1 ookinetes had a significantly lower average speed to that of IMC1h/GFP ookinetes (Figure 2B), as was reported for IMC1h null mutants (Tremp and Dessens, 2011). IMC1hΔ2 ookinetes also had a significantly lower average speed than IMC1h/GFP ookinetes, that was comparable to that of IMC1hΔ1 ookinetes (Figure 2B). The manner of ookinete movement as judged by their 3D trajectories was also examined: this showed that IMC1h/GFP ookinetes moved in a clearly helical fashion, while the trajectories of both IMC1hΔ1 and IMC1hΔ2 ookinetes were much more linear (Figure 2C). Similar differences were reported between wildtype and IMC1h null mutant ookinetes (Volkmann et al., 2012; Kan et al., 2014). Examination of videos of 3D ookinete movement showed that the abnormally shaped ookinetes of parasite lines IMC1hΔ1 and IMC1hΔ2 still possessed mildly helical movement albeit with a smaller radius, explaining why their trajectories appeared more linear (Videos S1–S3). These collective findings indicate that deletion of either domain 1 or domain 2 from IMC1h results in a loss-of-function phenotype with respect to ookinete motility.

We also assessed tensile strength of ookinetes using a hypo-osmotic shock assay. Hypo-osmotic conditions cause the cells to draw in water and swell, and the degree of hypo-osmotic stress a cell can tolerate is a measure of its tensile strength (Menke and Jockusch, 1991). In this assay, IMC1hΔ2 ookinetes were significantly more resistant to hypo-osmotic shock than their IMC1hΔ1 counterparts (Figure 2D), indicating that IMC1hΔ2 ookinetes have superior tensile strength to IMC1hΔ1 ookinetes. Thus, IMC1h lacking domain 2 is able to facilitate tensile strength above null mutant levels.

To assess the effects of the IMC1h module deletions on parasite infectivity, we infected *Anopheles stephensi* mosquitoes and recorded oocyst numbers as a measure of ookinete infectivity (Table 1). Reproducibly, both IMC1hΔ1 and IMC1hΔ2 ookinetes gave rise to statistically significantly (*P* < 0.05, *t*-test) reduced oocyst numbers compared to IMC1h/GFP control parasites (Table 1), indicating that they are less infective than their wildtype counterparts. IMC1hΔ2 ookinetes produced significantly higher oocyst numbers than IMC1hΔ1 ookinetes in one experiment (Experiment II, Table 1), indicating that they are more infective than their IMC1hΔ1 counterparts.

**Table 1:** Development of IMC1h mutant parasite lines in *Anopheles stephensi*.

**Experiment**	**Parasite line**	**Infection prevalence^a^**	**Mean ± SEM oocyst number^b^**	**Median oocyst number**	**Mean salivary gland sporozoite number^c^**	**Salivary gland sporozoites per oocyst^d^**
I	IMC1h/GFP	80 (15)	90 ± 19	82	n/a	n/a
	IMC1hΔ1	27 (15)	8.8 ± 5	5	n/a	n/a
	IMC1hΔ2	60 (15)	4.7 ± 1	4	n/a	n/a
II	IMC1h/GFP	100 (15)	102 ± 22	62	7,230 (13)	71
	IMC1hΔ1	100 (15)	7.9 ± 2.3	5	300 (20)	38
	IMC1hΔ2	100 (15)	32 ± 7.3	25	2,300 (20)	72
III	IMC1h/GFP	100 (21)	104 ± 25	48	n/a	n/a
	IMC1hΔ1	100 (21)	27 ± 6.5	20	625 (20)	23
	IMC1hΔ2	100 (22)	39 ± 10	26	2,760 (20)	70
IV	IMC1h/GFP	83 (24)	132 ± 26	85	7,968 (15)	60
	IMC1hΔ1	88 (24)	37 ± 7.3	19	528 (15)	14
	IMC1hΔ2	78 (27)	35 ± 6.8	20	2,496 (15)	71

a *Percentage of mosquitoes with at least one oocyst. (n) denotes the total number of mosquitoes analyzed*.

b *Oocysts were counted between 9 and 11 days post infection. Only infected insects were included*.

c *Average number of sporozoites per mosquito was calculated from (n) pooled salivary glands*.

d *Average number of sporozoites per mosquito divided by mean oocyst number*.

### Sporozoite-Specific Subcellular Localization of Mutant IMC1h::GFP and Cell Shape

**Figure 3:**
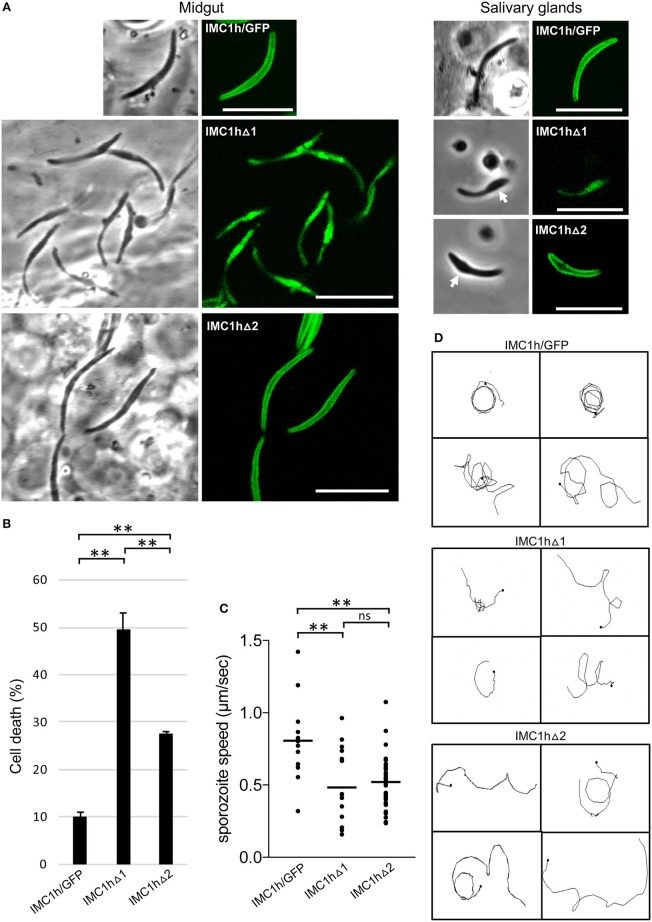
Sporozoite-specific subcellular localization of IMC1h::GFP in IMC1h mutants, and their phenotypes. **(A)** Confocal GFP fluorescence and brightfield images of sporozoites of IMC1h mutant parasite lines. Note that IMC1hΔ2 sporozoites display normal cortical localization of the GFP fusion protein and have normal shape in midguts, but abnormal shape with bulging area (white arrow) in salivary glands. Scale bar = 10 μm. **(B)** Percentage cell death after hypo-osmotic shock (0.33 × normal strength for 5 min). Cell death inversely correlates to tensile strength. ^*^statistically significant differences (*P* < 0.05). Error bars indicate S.E.M. values from two experiments. **(C)** Sporozoite speed expressed in μm/s. Images were captured every 10 s over a 2 min period. Plot is based on pooled data from two independent experiments and >60 sporozoites analyzed. Horizontal lines mark mean values. ^**^statistically significant differences (*P* < 0.005). ns, not significant. **(D)** Representative images of reconstructed 3D movement patterns from time-lapse movies of IMC1h mutants in Matrigel. The trajectories reveal the more meandering movement of IMC1hΔ1 and IMC1hΔ2 sporozoites compared to the more circular movement of IMC1h/GFP sporozoites.

Sporozoites of parasite line IMC1hΔ1 were misshapen possessing a bulging area (Figure 3A), similar to IMC1h null mutant sporozoites (Tremp and Dessens, 2011). Like IMC1hΔ1 ookinetes (Figure 2A), these sporozoites displayed only cytoplasmic fluorescence (Figure 3A) reflecting absence of SPN targeting. By contrast, the large majority (95%, *n* = 100) of IMC1hΔ2 midgut sporozoites had a normal crescent shape and displayed cortical localization of GFP fluorescence (Figure 3A) indicative of normal SPN targeting as observed in IMC1hΔ2 ookinetes (Figure 2A). Assessment of sporozoite sizes revealed that IMC1hΔ1 sporozoites had a significantly smaller average size than IMC1hΔ2 sporozoites (*P* < 0.001), which in turn were significantly smaller than IMC1h/GFP sporozoites (*P* < 0.005) (IMC1hΔ1 footprint: 6.9 ± 0.16 μm^2^; IMC1hΔ2 footprint: 8.5 ± 0.14 μm^2^; IMC1h/GFP footprint; 10.0 ± 0.42 μm^2^; *n* = 30). Interestingly, IMC1hΔ2 sporozoites lost their normal shape during transition from the midgut to the salivary glands, resulting in salivary gland sporozoites possessing a bulging area (Figure 3A). These combined observations indicate that IMC1hΔ2 parasites possess an intermediate phenotype with respect to sporozoite morphogenesis.

### Tensile Strength, Motility, and Infectivity IMC1h Mutant Sporozoites

Tensile strength assessment of midgut sporozoites indicated that IMC1hΔ2 sporozoites had higher tensile strength than their IMC1hΔ1 counterparts, but lower tensile strength than IMC1h/GFP sporozoites (Figure 3B). Both IMC1hΔ1 and IMC1hΔ2 sporozoites had similar average speed that was significantly reduced compared to that of their IMC1h/GFP counterparts (Figure 3C). The manner of sporozoite 3D movement as judged by their trajectories in Matrigel was also different between the parasite lines examined: while IMC1h/GFP sporozoites moved in a predominantly circular fashion in Matrigel, the trajectories of IMC1hΔ1 and IMC1hΔ2 sporozoites were more meandering and less circular (Figure 3D), as indeed was reported for IMC1h null mutants (Volkmann et al., 2012). These collective observations indicate that deletion of either domain 1 or domain 2 from IMC1h results in a loss-of-function phenotype with respect to sporozoite motility.

Sporozoite infectivity to the mosquito was assessed by counting salivary gland sporozoite numbers 3 weeks post-infection. Assuming a linear correlation between the number of oocyst and the number of sporozoites produced, these data indicated that IMC1hΔ2 sporozoites were almost three times more infective to salivary glands than IMC1hΔ1 sporozoites (*P* < 0.05, *t*-test), and had similar efficacy to IMC1h/GFP sporozoites in colonizing the salivary glands (Table 1). This is consistent with previous findings that IMC1h null mutant sporozoites are less invasive than their wildtype counterparts (Tremp and Dessens, 2011; Volkmann et al., 2012). Despite their presence in salivary glands, we failed to transmit IMC1hΔ1 and IMC1hΔ2 sporozoites by mosquito bite to naive mice, indicating that their infectivity to the mammalian host by natural route of transmission is compromised.

## Discussion

*Plasmodium* alveolins have important roles in morphogenesis, tensile strength and motility of the zoite stages, and in many cases their disruption leads to loss of parasite fitness, infectivity and transmission (Khater et al., 2004; Tremp et al., 2008, 2014; Tremp and Dessens, 2011). Here, we present data from a structure-function analysis of the alveolin IMC1h, aimed to determine the contributions of its “alveolin” and “non-alveolin” modules to protein function. This was based on a research strategy of allelic replacement of *imc1h* with modified versions of the gene fused to a GFP module for localization purposes. Our results demonstrate that the alveolin module of IMC1h is necessary for targeting the protein to the cortical membrane skeleton. This is in full agreement with a report showing that the alveolin domain of TgIMC3 can target YFP to the SPN of *Toxoplasma gondii* in a wildtype parasite background (Anderson-White et al., 2011). Our data show furthermore that the alveolin domain of IMC1h is sufficient to increase tensile strength above null mutant levels. Given the filamentous nature of the SPN (Mann and Beckers, 2001), the assembly or incorporation of the alveolins into such structures is likely to be a prerequisite for the provision of mechanical strength. Hence, our findings strongly suggest that the alveolin module contains the properties for intermediate filament formation, consistent with the fact that it is found in all alveolins and indeed is the distinguishing feature of this protein family. The processes of filament formation and SPN recruitment could be mechanistically linked as proposed (Tremp et al., 2017).

Previous studies have shown that knockout of IMC1a, IMC1b, or IMC1h reduced motility and tensile strength at the same time (Khater et al., 2004; Tremp et al., 2008; Tremp and Dessens, 2011). For this reason, it has not been previously possible to dissect the individual contributions of tensile strength and motility to zoite infectivity. This study shows that zoites of parasite line IMC1hΔ2 are better at infecting mosquito tissues than the equivalent IMC1hΔ1 parasites (Table 1). As IMC1hΔ1 and IMC1hΔ2 zoites do not differ discernibly in their motilities, their differences in tensile strength are likely to be the cause of their distinct invasive capacities. These experiments thus show for the first time that tensile strength is an important contributor to zoite infectivity in the mosquito. Cell rigidity and flexibility are likely to be important when ookinetes escape the blood meal in the midgut lumen and cross the peritrophic matrix and midgut epithelium, a process that has been described to cause major cell constrictions (Vernick et al., 1999; Han et al., 2000), and the same can be envisaged in sporozoites when entering mosquito salivary glands.

IMC1hΔ2 sporozoites are larger than IMC1hΔ1 sporozoites and smaller than wildtype sporozoites, and they display normal shape when first formed. These observations indicate that IMC1h without its non-alveolin module causes an intermediate phenotype with regards to sporozoite morphogenesis. Interestingly, mutations of putative amino- or carboxy-terminal palmitoylation sites of the sporozoite-specific alveolin IMC1a have also been shown to affect sporozoite shape and size, being in between that of null mutant and wildtype sporozoites (Al-Khattaf et al., 2017). Collectively, these findings point to a fundamental role of the alveolins in determining sporozoite size and shape. The mechanisms by which alveolins participate in zoite morphogenesis remain poorly understood and, interestingly, knockouts of other SPN proteins that are structurally unrelated to alveolins such as G2 and PhIL1 cause similar cell shape abnormalities (Barkhuff et al., 2011; Tremp et al., 2013). Zoite morphogenesis is concurrent with the formation of the IMC and SPN structures (Hu et al., 2002; Tremp et al., 2008), and one possibility is that this is a highly constrained process that is very sensitive to structural disruption of any SPN component.

The apparent lack of correlation between motility and tensile strength (Figures 2, 3) indicates that these two processes are in fact mechanistically uncoupled. This corroborates previous findings that IMC1h null mutant ookinetes have comparably reduced speed to similarly misshapen G2 null mutant ookinetes, despite these two parasite lines possessing different tensile strengths (Tremp et al., 2013). Thus, these observations support a role of IMC1h in motility through interactions with the motility apparatus, possibly via IMC-resident bridging proteins, rather than by simply contributing mechanical support to the IMC. Furthermore, our finding that IMC1hΔ2 parasites fail to rescue the IMC1h null mutant phenotype with respect to motility points to an involvement of the non-alveolin domain of IMC1h in the motility process. We therefore postulate that domain 2 is the part of IMC1h that engages with the motility apparatus, while the alveolin module is primarily involved in filament formation, SPN recruitment and viscoelasticity. Comparative alveolin interactome studies using IMC1hΔ2, IMC1hΔ1, and IMC1h/GFP ookinetes are underway to test this hypothesis and identify candidate IMC proteins interacting with domain 2. In this context, it is also worth noting that the other two alveolins with demonstrated roles in motility: IMC1a and IMC1b, also possess a conserved carboxy-terminal domain that has no structural relationship to the alveolin module (Khater et al., 2004; Tremp et al., 2008), and which may fulfill a similar role in motility to domain 2 of IMC1h.

Both ookinetes and sporozoites possess chirality, which is thought to be responsible for the circular nature of their directional movement (Kudryashev et al., 2012; Kan et al., 2014). During circular forward movement in Matrigel, ookinetes also rotate on their axis resulting in a helical, corkscrew-like 3D trajectory. By contrast, sporozoites possess dorsoventral polarity (Kudryashev et al., 2012) and helical movement is largely missing, explaining their predominantly circular directional movement in both 2D and 3D environments. Previous studies showed that the simultaneous absence of the alveolins IMC1h and IMC1b did not further affect ookinete cell shape, but further reduced ookinete speed and tensile strength compared with the respective single mutants, indicating that these two alveolins operate autonomously and contribute to motility independently of cell shape (Tremp and Dessens, 2011). We show here that the deletion of domains 1 or 2 from IMC1h not only reduces ookinete and sporozoite speed, but also alters their 3D movement to a markedly more linear fashion compared to that of their counterparts expressing full-length IMC1h. Nonetheless, examination of IMC1h mutant ookinetes reveals that the underlying motion remains mildly helical (Videos S1–S3), indicating that the biomechanics of movement have remained fundamentally the same as those of normal-shaped ookinetes. Changes in ookinete shape resulting from IMC1h knockout reduce the level of chirality and it was proposed that this determines the way in which these cells move in a 3D environment (Kan et al., 2014). The same could apply to the IMC1h mutant zoites described here. Consolidating the entire spectrum of motility observations, a model is emerging whereby zoite speed is largely cell shape-independent, whilst 3D movement is affected by the shape of the cells.

Salivary gland sporozoite numbers indicate that the infectivity of IMC1hΔ2 sporozoites to the mosquito is higher than that of IMC1hΔ1 sporozoites (Table 1), despite both mutant sporozoite populations possessing similar motility defects. These observations suggest that motility makes a relatively minor contribution to the sporozoites' invasive power. This is consistent with findings that IMC1h null mutant sporozoites have similar *in vitro* hepatoma cell transmigration and infection rates to their wildtype counterparts, despite having significantly reduced speed (~ 2-fold) and more meandering movement patterns (Volkmann et al., 2012). Heat shock protein 20 null mutant sporozoites, which can move only very slowly, are also able to invade hepatocytes at the same efficiency as wild-type sporozoites, indicating that at least rapid gliding is not essential for efficient invasion (Montagna et al., 2012). The invasive power of IMC1hΔ2 sporozoites with regards to salivary glands was however not reflected in a greater infectivity to the mouse, as we were repeatedly unable to transmit these parasites by the normal route of mosquito bite. IMC1h null mutants are not naturally transmissible, but are infective to mice when injected intravenously (Volkmann et al., 2012). These collective observations thus identify traversal of the dermis as a major bottleneck for sporozoite infection of the mammalian host.

## Data Availability

All datasets generated for this study are included in the manuscript and/or the Supplementary Files.

## Ethics Statement

This study was carried out in accordance with the Laboratory Animal Science Association guidelines. All laboratory animal work was approved by the Animal Welfare and Ethical Review Board of the London School of Hygiene and Tropical Medicine, and by the United Kingdom Home Office. Work was carried out in accordance with the United Kingdom Animals (Scientific Procedures) Act 1986 implementing European Directive 2010/63 for the protection of animals used for experimental purposes.

## Author Contributions

JD and CV contributed conception and design of the study. MC, AT, SS, and JD performed experiments and interpreted results. MC wrote the first draft of the manuscript. All authors contributed to manuscript revision, read, and approved the submitted version.

### Conflict of Interest Statement

The authors declare that the research was conducted in the absence of any commercial or financial relationships that could be construed as a potential conflict of interest.

## Supplementary Material

Video S1 Time lapse movie of a representative IMC1h/GFP ookinete in Matrigel.

Video S2 Time lapse movie of a representative IMC1hΔ1 ookinete in Matrigel.

Video S3 Time lapse movie of a representative IMC1hΔ2 ookinete in Matrigel.
